# Electronic and manual registration of Manchester System: reliability, accuracy, and time evaluation*

**DOI:** 10.1590/1518-8345.3170.3241

**Published:** 2019-12-05

**Authors:** Emilia Aparecida Cicolo, Heloísa Helena Ciqueto Peres

**Affiliations:** 1 Universidade de São Paulo, Hospital Universitário, São Paulo, SP, Brazil.; 2 Universidade de São Paulo, Escola de Enfermagem, São Paulo, SP, Brazil.

**Keywords:** Nursing, Triage, Decision Support Systems, Clinical, Computers, Informatics, Nursing Informatics, Enfermagem, Triagem, Sistemas de Apoio a Decisões Clínicas, Computadores, Informática, Informática em Enfermagem, Enfermería, Triaje, Sistemas de Apoyo a Decisiones Clínicas, Computadores, Informática, Informática Aplicada a la Enfermería

## Abstract

**Objective:**

to evaluate the degree of reliability, accuracy and timing to perform the Manchester Triage System in electronic and manual records.

**Method:**

exploratory-descriptive research. Case series corresponded to a total of 20 validated simulated clinical cases applied to a sample of 10 nurses. For data collection each participant received 4 clinical cases in 2 different phases of the study, using manual and electronic registration. The variables related to the triage were: incomplete data filling, discriminator, flowchart, priority level, vital signs and triage timing.

**Results:**

moderate reliability for choosing flowcharts and substantial reliability for determining discriminators in both records; substantial and moderate, for priority, respectively, in manual and electronic registration. For vital signs, it was weak in manual recording and substantial in electronic. Accuracy showed a statistically significant difference related to vital signs. The average timing on triage was shorter with the use of electronic registration.

**Conclusion:**

the use of electronic registration has advantages regarding reliability, accuracy and timing to perform the triage, pointing to the importance of adopting technologies in the management and care work process in health services.

## Abstract (pt)

**Objetivo:**

Avaliar o grau de confiabilidade, acurácia e tempo despendido para realização do Sistema Manchester de Classificação de Risco em registros eletrônico e manual.

**Método:**

exploratório-descritivo. Casuística do estudo correspondeu ao total de 20 casos clínicos simulados validados aplicados para amostra de 10 enfermeiros. Para coleta de dados cada participante recebeu 4 casos clínicos em 2 diferentes fases do estudo, com uso em registro manual e eletrônico. As variáveis relacionadas à classificação de risco foram: preenchimento incompleto dos dados, discriminador, fluxograma, nível de prioridade, sinais vitais e tempo despendido com classificação de risco.

**Resultados:**

Confiabilidade moderada para escolha dos fluxogramas e substancial para determinação dos discriminadores em ambos os registros; substancial e moderada, para prioridade, respectivamente, no registro manual e eletrônico. Para sinais vitais, apresentou-se fraca no registro manual e substancial no eletrônico. A acurácia apresentou diferença estatisticamente significante relacionada aos sinais vitais. A média de tempo despendido com a classificação de risco foi menor com utilização do registro eletrônico.

**Conclusão:**

O uso do registro eletrônico apresenta vantagens referentes à confiabilidade, acurácia e tempo despendido para a realização da classificação de risco, indicando a importância da adoção de tecnologias no processo de trabalho gerencial e assistencial nos serviços de saúde.

## Abstract (es)

**Objetivo:**

evaluar el grado de confiabilidad, precisión y tiempo utilizado para realización del Sistema Manchester de Clasificación de Riesgo en registros electrónico y manual.

**Método:**

exploratorio y descriptivo. La casuística del estudio correspondió al total de 20 casos clínicos simulados, validados y aplicados en una muestra de 10 enfermeros. Para la recolección de datos cada participante recibió 4 casos clínicos, en 2 diferentes fases del estudio, utilizando registro manual y electrónico. Las variables relacionadas a la clasificación de riesgo fueron: llenado incompleto de datos, discriminador, diagrama de flujo, nivel de prioridad, señales vitales y tiempo utilizado en la clasificación de riesgo.

**Resultados:**

confiabilidad moderada para elección de los diagramas de flujo y substancial para determinación de los discriminadores en los dos tipos de registro; substancial y moderada para prioridad, respectivamente, en el registro manual y electrónico. Para señales vitales, se presentó débil en el registro manual y substancial en el electrónico. La precisión presentó diferencia estadística significativa relacionada a las señales vitales. La media de tiempo utilizado con la clasificación de riesgo fue menor con la utilización del registro electrónico.

**Conclusión:**

el uso del registro electrónico presenta ventajas referentes a la confiabilidad, precisión y tiempo utilizado para la realización de la clasificación de riesgo, indicando la importancia de la adopción de tecnologías en el proceso de trabajo administrativo y asistencial en los servicios de salud.

## Introduction

Overcrowding of emergency services is a worldwide phenomenon constantly shown in different sorts of media^(^ 1 ^-^ 2 ^)^.

In Brazil, the increasing demand for hospital care has harmful consequences for health service users, such as a long waiting for medical care. And triage, based on triage systems, is effective in managing the flow of patients in emergency services, improving this situation^(^ 2 ^-^ 6 ^)^.

The most widely used system in Brazil is the Manchester Triage System (MTS). From a decision-making process, the healthcare professional defines the individual’s complaint and selects a flowchart, that is, set of signs and symptoms in the form of structured questions (discriminators). It may also be necessary to check vital signs (temperature, oximetry, heart rate, blood glucose) and to apply scales (Glasgow coma, pain) so that at the end of triage the priority level corresponding to the maximum time for medical care is determined^(^ 7 ^)^.

There are five levels of priority: immediate (red) 0 minutes, very urgent (orange) 10 minutes, urgent (yellow) 60 minutes, standard (green) 120 minutes, non-urgent (blue) 240 minutes and (white) intended for events, situations or complaints not compatible with the emergency service^(^ 7 ^)^.

The Manchester system contributes to emergency system management, offers better working conditions by implementing horizontal care and increases user satisfaction with high priority levels^(^ 7 ^-^ 10 ^)^.

 Triage can be performed from manual or electronic registration. Manual registration are the most traditional forms, but there are risks of misplacement and greater difficulty in retrieving information. Electronic registration allow to deal with large amount of complex information in an organized and fast way, being important tools for the health information system^(^ 11 ^-^ 12 ^)^.

The use of electronic registration, based on decision support systems (DSS), supports clinical reasoning by providing guidelines, reminders and alert signs to health professionals during patient care, which may represent advantages to the triage process^(^ 13 ^)^.

The Triage of the University Hospital of the University of São Paulo (HU-USP) uses the MTS software and, when not possible its use, such as power outages or problems in the institution’s computer department, manual registration is adopted. In this scenario, it was possible to observe more time spent in the process and incomplete filling of classification data, as well as discontent among the workers in the unit using manual registration, because of the need to check the guides and manuals and the for the absence of alert signs and structured and logical structure of the data, present in the electronic registration, for decision making.

These experiences allowed the reflection on the use of electronic health records and the competence of nurses in the use of data and information for clinical decision making, emerging interest in investigating this theme.

In order to compare the different types of records in triage, the authors adopted the concepts of reliability and accuracy. Also, the timing for triage was considered.

According to the international consensus for defining the measurement properties of instruments, the Consensus Based Standards for the Selection of Health Measurement Instruments (COSMIN) initiative, reliability refers to the maintenance of scores in repeated measurements in different situations, without changes compared to patients and free from measurement errors. Evaluations can be performed at the same time, either with a time interval (test-retest) and by the same person (intra-evaluator) or by different evaluators (inter-evaluator)^(^ 14 ^)^.

Accuracy or exactness is a type of measurement that determines the degree of agreement between the measured result and the true value^(^ 15 ^-^ 17 ^)^. In studies on triage, the accuracy represents the accuracy of the evaluators’ responses in relation to the gold standard on: flowchart, discriminator, priority and vital signs^(^ 5 ^,^ 16 ^)^.

Despite the importance of this theme, it is also noted that from a systematic review^(^ 18 ^)^ no studies were found that compared the reliability, accuracy or timing of MTS when using manual and electronic records, highlighting the gap of knowledge. Thus, the question was: What is the degree of reliability and accuracy with the use of electronic and manual registration in the application of MTS? What is the timing to perform triage using electronic and manual registration?

The following hypotheses were raised: There are no differences in the degree of reliability and accuracy between either electronic or manual registration in the application of MTS. The triage timing in electronic registration is shorter the than in the manual registration.

The objective of the study was to evaluate the degree of reliability, accuracy and timing using the Manchester Triage System in electronic and manual registration.

## Method

This is an exploratory-descriptive research, approved by the Research Ethics Committees of the University of São Paulo School of Nursing, consubstantiated opinion n. 1,915,863 and identifier CAAE 61685516.1.0000.5392 and of the University Hospital of the University of São Paulo (HU-USP), opinion n. 1,969,690 and CAAE identifier 61685516.1.3001.0076, and to ensure the quality of work the criteria established by SQUIRE 2.0 (Revised Standards for Quality Improvement Reporting Excellence) were adopted^(^ 19 ^)^.

The study was conducted in the HU-USP Triage between April and June 2017. The HU-USP is a general public teaching hospital of secondary complexity in city of São Paulo, with 206 beds registered at the Health Service Offering Regulation Center (CROSS) and is part of one of the most important teaching institutions in Latin America^(^ 20 ^-^ 21 ^)^.

This unit uses the MTS for the early assessment of patients seeking emergency care at the institution, except pregnant women with obstetric complaints and patients who arrive by ambulance or who have an evident emergency.

The Triage is located near the hospital’s entrance for patients and it works, according to the patients demand, from Monday to Sunday from 7 am to 7 pm. There are 3 nursing offices and 7 medical offices, and the triage is done exclusively by nurses. It is worth clarifying that in other working hours, the service is provided by the doctor in order of arrival and according to specialties, given the low patients inflow.

All nurses from triage, as well as part of the nurses from adult emergency room, children’s emergency room and outpatient unit, some chiefs of nurses and physicians took the MTS classifier course. However, the nurse is the health professional who does the triage.

This way, the population consisted of 43 (100%) nurses from HU-USP who were referred by the hospital to take the MTS classifier course of the Brazilian Group of Risk Classification (GBCR in Portuguese) and were approved.

Those who, during data collection, were not on the institution (on dismissal, leave or vacation) and the researcher herself were excluded from the study.

After applying these criteria, 5 people were excluded, with 38 nurses remaining. The opportunity selection process was performed according to simple systematic random probability sampling through electronic draw.

The amount of cases and nurses were determined from calculations performed in the R 3.3.0 system with the irr package, to obtain a Cohen kappa coefficient greater than or equal to 0.5, 95% confidence and test power of 80%, considering the number of patients classified at each clinical priority level at HU-USP in 2016, that is, 4800 (60%) green, 1440 (18%) yellow, 1120 (14%) blue, 4% (320) orange, 3.5% (280) white and 0.5% (40) red*.

A minimum sample of 19 simulated cases to be distributed to a minimum of 5 participants was estimated. For a fair distribution, a sample of 20 simulated clinical cases and 10 nurses was adopted, with 4 cases for each of them.

The study sample corresponded to a total of 20 simulated clinical cases applied to a sample of 10 nurses. Each participant received 4 clinical cases in 2 different phases of the study.

In this way, the clinical cases used were requested to GBCR by this group in the classifier training courses in Brazil and which had been evaluated by specialists. According to an agreement between the group’s representatives, the researcher and the advisor, the Brazilian Group of Risk Classification provided 37 simulated clinical cases, subject to confidentiality.

Twenty simulated clinical cases were selected from the analysis of the researcher and the advisor, who did the exhaustive reading of all cases and proceeded to the selection, adopting as criteria the distribution of patients treated at the HU-USP and the maintenance of a heterogeneity as to the distribution of cases by clinical priority.

The clinical cases presented involve conditions that simulate triage, in which data on patient identification (such as gender and age), clinical complaints and vital sign values ​​are presented. Responses submitted as correct by the GBCR were adopted as the gold standard.

Since the implementation of MTS, HU-USP has been using Trius^®^ for triage. This device contains Emerges® software, which has all the Manchester system flowcharts, enabling to check vital signs and enter them directly into the computer.

The simulated clinical cases were given out in printed form and divided among the research participants upon an Excel^®^ drawing of the pairs of individuals.

Data collection was performed in 2 phases, using manual and electronic registration. In phase 1, after signing the Informed Consent Form (ICF), the participant filled out the “Characterization of the Population” questionnaire, to survey the socio-demographic profile and computer abilities and to classify the manual registration of the 4 clinical cases. In phase 2, after an approximate interval of 4 weeks, nurses would perform the triage using Emerges® on the same 4 clinical cases.

The time between the two phases of data collection was determined considering studies on triage, which applied clinical cases at two different moments^(^ 22 ^-^ 23 ^)^. There is no consensus in the literature about the optimal timing; however, this should not be too long or too short. Long periods favor the acquisition of new learning and short periods may be influenced by the memory effect^(^ 24 ^)^.

The nurses were invited by the researcher to participate in the study in their work units and if they agreed to participate in the research, they were instructed to: Sign the Informed Consent Form (ICF), fill out the “Characterization of the Population” questionnaire, which aimed to the survey of the socio-demographic profile and computer ability and to manually classify the 4 simulated clinical cases. To do so, they would use 4 triage forms developed by the researcher, based on the form used by HU-USP itself in situations where the triage is required manually. In these forms, there were blanks to fill in with information regarding the triage, including the start and ending time, which should be filled out by each subject.

Study participants were instructed to fill out all forms at their workplace by themselves and check only to the book with the MTS protocol, as is the case in real-world triage. In addition, the deadline for returning the forms was agreed.

After the 4-week interval, the researcher would contact the nurses again to schedule with each participant a date of their availability for further research using the electronic registration. At this stage, during working hours, each participant went to the HU-USP computer department to perform the triage from the electronic register. This process was performed individually and the SMCR’s book was not included, as it is in the institution’s Triage.

The population characterization variables analyzed were: gender, age, highest degree, current sector, current sector experience, previous sector, previous sector experience, year of SMCR classifier course , experience with the use of electronic and/or manual registration in the application of SMCR, average time of daily use of the computer in general, main use of the computer (work, study and leisure) and level of computer ability. These data were described in absolute frequencies and percentages.

The variables related to the triage were: incomplete data, discriminator, flowchart, priority level, vital signs and triage timing. These data were compared inter-rater in relation to the gold standard.

To calculate reliability, Cohen’s kappa coefficient was used, which has agreement values ​​divided into different levels: < 0 (no agreement); 0.01 - 0.20 (slight); 0.21 - 0.40 (weak); 0.41 - 0.60 (moderate); 0.61 - 0.80 (substantial) and 0.81 - 1.00 (perfect)^(^ 25 ^)^.

Accuracy was determined by comparing the inter-rater responses with the gold standard by the percentage of agreement between them, adopting a 95% confidence interval and a p-value less than or equal to 0.05, according to the Pearson’s Chi Square test.

For the analysis of the triage timing, the Wilcoxon-Mann-Whitney test was used, with a 95% confidence interval and p value less than or equal to 0.05.

## Results

Regarding the characterization of the sample, the average age of the participants was 38.7 years. The other results are presented in Table 1.

**Table 1:** Sample characterization. Sao Paulo, SP, Brazil, 2018

Variable	n
Gender	
Female	9
Male	1
Highest degree	
Specialization	5
Master	5
Current unit	
Triage	3
Adult emergency room	3
Children’s emergency room	2
Outpatient unit	1
Surgery room	1
Previous unit	
Adult emergency room	3
None	2
Triage	1
Medical unit	1
Transport	1
Training and quality service	1
Hygiene	1
Year of classifier course	
2012	4
2013	1
2014	1
2015	2
2016	1
Does not know	1
Triage registration type	
Electronic	7
Manual	3
Use of computer*	
Work	7
Study	5
Leisure	1
Informatics competence	
Basic	1
Informational	8
Information management	1

* There are more than 1 answers per participant

It can be noted that most participants are female, have previous experience in triage and/or emergency room and have already used the electronic registration.

The average time working in the units was 7.6 years in the current unit and 6.5 years in the previous one. The average time of computer use was 4.2 hours daily.

Regarding missing data, patient identification data, beginning and end of triage and physician referral were recorded in all triages performed. Regarding the triage variables, there was a lack of records regarding priority (phase 1) and vital signs (in both phases).

Reliability data are shown in Table 2.

**Table 2:** Distribution of inter-rater reliability according to the variables in phases 1 and 2 of the study. Sao Paulo, SP, Brazil, 2018

Variable	Phases of the Study	Kappa value
Discriminator	1	0.633
	2	0.788
Flowchart	1	0.580
	2	0.423
Priority	1	0.703
	2	0.454
Vital signs	1	0.239
	2	0.675

The values ​​are similar in the discriminator and flowchart variables; however, they present greater variation in priority and vital signs. Concerning the priority, the agreement was higher in the manual registration, and, for vital signs, the value was higher in the electronic registration.

For accuracy, as shown in table 3, there was no statistically significant difference regarding the choice of discriminator, flowchart and priority; However, when analyzing vital signs, a statistically significant difference was observed in relation to the number of correct answers.

**Table 3:** Accuracy distribution according to the variables in phases 1 and 2 of the study. Sao Paulo, SP, Brazil, 2018

Attributes	Accuracy	Phase 1		Phase 2		p-value
		N	%	N	%	
Flowchart	Correct	30	75.0	27	67.5	0.4586
	Incorrect	10	25.0	13	32.5	
Discriminator	Correct	20	50.0	21	52.5	0.8230
	Incorrect	20	50.0	19	47.5	
Vital signs	Correct	9	22.5	24	60.0	< 0.001
	Incorrect	31	77.5	16	40.0	
Priority	Correct	29	72.5	30	75.0	0.7994
	Incorrect	11	27.5	10	25.0	

From Table 4, it is highlighted the insufficient registration of vital signs in both phases of the study.

**Table 4:** Distribution of inter-rater responses in relation to the gold standard in recording vital signs in phases 1 and 2 of the study. Sao Paulo, SP, Brazil, 2018

Inter-rater responses:	Phase 1		Phase 2	
Vital signs	N	%	N	%
Different	3	10%	0	0%
None	1	3%	0	0%
Missing vital signs	17	55%	11	69%
Exceeding vital signs	10	32%	5	31%
Total	31	100%	16	100%

Regarding priority errors, it was observed that in most cases higher priority levels of triage were considered, as shown in Table 5.

**Table 5:** Distribution of inter-rater responses in relation to the gold standard in determining priority in phases 1 and 2 of the study. Sao Paulo, SP, Brazil, 2018

Attribute	Phase 1		Phase 2	
Priority	N	%	N	%
Higher	7	64%	8	80%
Lower	3	27%	2	20%
Blank	1	9%	-	-
Total	11	100%	10	100%

As for the timing, it should be noted that for the manual registration, the average was 3.179 minutes, and for the electronic registration, it was 2.425 minutes (p value: 0.0023), constituting a statistically significant difference.

## Discussion

The profile of nurses in this study is similar to that presented in the research “Nursing Profile of Brazil”, where most nurses are 357,551 (86.2%) female, 263,687 (63.6%) aged between 31 and 55 years old and 332,028 (80.1%) with a postgraduate course *lactu* or *strictu* *sensu* (80.8%)^(^ 26 ^)^.

In this study, most nurses^(^ 7 ^)^ have already worked on triage and have used manual and electronic registration, being a facilitator to perform the classification^(^ 27 ^)^. As professional experience helps in identifying patient needs and determining the priority established for care. In addition, allied to the intuitive ability of nurses, is responsible for personal and specific knowledge on this subject.

The milestone of the HU-USP triage was in 2012, with the offering of classifier courses to the institution’s professionals. This period meets a larger number of research participants (4) who took the course.

Regarding computer use, 7 nurses have as the main purpose of their work, on average 4.22 hours daily, the use of this equipment. Thus, it can be inferred that the average daily hours using this equipment was mainly intended for work activities. This finding corroborates the data from the research “Information and Communication Technologies (ICTs) Health 2016” which showed high availability of computers to nurses in health facilities in Brazil. A total of 1919 (88%) professionals have at least 1 equipment available at their workplace and 1265 (58%) nurses always use it^(^ 13 ^)^.

The use of computers by nurses in health services stands out as a tool that organizes, assists, speeds up and humanizes nursing care^(^ 28 ^)^.

Nurses, due to their historical role of mediators between the patient and the health system, are increasingly using electronic records as a health work tool to provide support for patient care and clinical and managerial decision making. in nursing, which justifies the mastery of these abilities^(^ 29 ^)^.

Regarding the knowledge on computer by the nurses in the study there was a homogeneity. Most^(^ 8 ^)^ considered having knowledge at least at the informational level and only one person had information management level.

This variable was analyzed from the definitions of nursing informatics competences from the TIGER (Technology Informatics Guiding Education Reform) initiative. The Basic competency refers to the concepts of information and communication technology (ICT), computer use and file development, use of the Internet. Informational competence is the ability to identify the information necessary for a specific purpose, to locate, evaluate and correctly apply the relevant information. Information management competence encompasses the process of collecting, processing, presenting and communicating data as information or knowledge^(^ 30 ^-^ 31 ^)^.

Considering this definition of nursing informatics competence related to triage on electronic registration, the requirement of competence beyond the simple use of systems and computers is central, relating more and more to the impact of information and information management as a health service management tool.

Nurses need to expand and develop nursing informatics skills as there is an evolution of informatics in health care, considering it as a strategic management resource that can contribute to the quality, efficiency and effectiveness of patient care. Nurses, too, need to know how to use information systems to develop and employ an empirical knowledge base for nursing practice, contributing to the broad structure of clinical research necessary for patient care and the protection and improvement of nursing care on population’s health^(^ 29 ^)^.

However, data from the (ICT) Health 2016 survey showed that only 567 (26%) of professionals affirm to participate in ICT training, even though most 1875 (86%) understand that the use of electronic systems improves efficiency of care^(^ 13 ^)^.

As for study reliability, the hypothesis was confirmed for the choice of flowcharts and discriminators with the use of electronic and manual records and showed differences in determining priority and recording vital signs in the application of MTS.

From the analysis of inter-rater kappa values ​​in the manual and electronic registration, there was no difference regarding the choice of flowchart and discriminator. For the priority variable, there was a difference in inter-rater agreement with the use of manual and electronic registration, being substantial and moderate, respectively. Concerning vital signs, agreement was poor in the use of manual and substantial electronic registration.

Differences in inter-rater reliability were not expected, as the electronic and manual records are only resources used for MTS application and, therefore, the results would not vary depending on the adopted registration type.

However, there are studies, which also present similar values ​​to this study, regarding the inter-rater reliability in determining the flowcharts and discriminators with the use of the electronic registration. In this research, the data found are substantial in the choice of flowchart (kappa 0.66) and moderate regarding the discriminators (kappa 0.47)^(^ 32 ^)^.

To determine the causes of the difference in inter-rater priority determination, a more detailed analysis of each of the triage and the correlations between its characteristics and raters is required.

Other studies have shown differentiation as to the priority variable and the ways of application of the MTS. The agreement obtained ranged from weak (kappa 0.27) to substantial (kappa 0.63) for manual registration, and was moderate (kappa 0.53) to perfect (kappa 0.83) for electronic registration^((32-34 ))^. The findings of the present study are in these same value ranges.

Publications about other triage systems, also, showed similar values. In Canadian Triage and Acuity Scale (CTAS) studies, agreement was moderate with manual registration (kappa 0.51) and ranged from moderate to substantial (kappa 0.40 to 0.75) with electronic registration (35 -37). In a study conducted with the Soterion Rapid Triage System, the authors obtained a perfect agreement (kappa 0.87) with the use of electronic registration^(^ 38 ^)^.

Despite the differences in agreement values ​​for determining priority with the use of manual and electronic registration, the values ​​obtained reached a minimum moderate level. In addition, it is not possible to state that one way of execution is superior to the other and these data cannot be analyzed in a single way, and it is necessary to consider the correctness rates in relation to the gold standard, that is, the accuracy.

The greater inter-rater agreement in the registration of vital signs, with the use of electronic registration, may be due to alert barriers, which point out the vital signs that must be measured in each corresponding flowchart and support nurses’ decisions, avoiding forgetfulness and excess on data registration.

Analyzing the fulfillment of vital signs is important for assessing the triage, as unchecked vital signs may hide changes in the patient and over-measuring vital signs may depict more time spent.

Thus, the relevance of this research stands out, given these findings and the lack of publications on the reliability of triage systems that consider the filling of vital signs as a variable.

Regarding accuracy, the hypothesis for choosing the flowchart, discriminator and priority in the two phases of the study was confirmed, being in the same range of values ​​presented in studies on MTS and electronic registration. A statistically significant difference was observed only for the vital signs attribute.

Other publications presented, for the choice of flowchart, values ​​between 64% and 73.5%; as for the discriminator, the results were between 28% and 58.6%; and in relation to priority, they ranged from 66% to 77.6%^(^ 32 ^,^ 34 ^)^. It is noteworthy the lack of studies on the MTS and manual registration that have performed these calculations.

In a study on the Pediatric Canadian Triage and Acuity Scale (PedCTAS), a pediatric triage system, no statistically significant differences were found between the electronic and manual records in determining priority. The agreement between the nurses and the gold standard obtained values ​​equal to 57% in the manual registration and 55% in the electronic one^(^ 37 ^)^.

Despite the high agreement between the raters and the gold standard regarding priority in manual and electronic records, errors related to this variable can result in harm to patients and emergency services. And when analyzing these cases where errors occurred, it was found that most were due to overtriage.

In cases of overtriage, excessive resources are shifted to patients with non-emergent problems, resulting in increased costs and delayed care of the most severely ill patients^(^ 39 ^-^ 40 ^)^.

In undertriage, the most severe patients would take longer to be seen by the doctor, which could lead to complications to their health^(^ 39 ^-^ 40 ^)^.

Analyzing the types of errors related to the registration of vital signs, it is noted that most were denoted by failing to register one particular vital sign. A study on the use of an institutional triage protocol with manual registration also found problems regarding the registration of vital signs. In 221 (58%) cases, no vital signs were recorded; however, in this institution, the parameters are measured by the nursing technician before the classification^(^ 5 ^)^.

The electronic registration warning barriers may have contributed to the errors found in the present study, because besides avoiding forgetting and registration excess signals, they compare the values ​​registered to the normality standards, preventing the continuity of the triage if there are abnormalities in values.

However, it is important to highlight that the clinical reasoning of nurses still prevails. Professionals need to interpret these alerts presented by the electronic record. In some situations, for example, vital signs may be recorded above or below normal due to sensor positioning problems. Thus, the barriers should only act as alerts, prevailing the clinical reasoning of nurses.

In the present study, it was found that in the registration of vital signs, errors with the use of electronic registration originate from the incorrect choice of flowchart and/or discriminator. This happens because of vital signs varies according to the selected flowcharts and discriminators.

In general, it is noted that the use of electronic registration reduced the occurrence of errors in relation to the registration of vital signs, that is, in a larger number of cases, vital signs were recorded as determined by the corresponding flowcharts.

Thus, computerization contributed so that all possible changes in vital signs corresponding to flowcharts could be verified, avoiding the cases of overtriage and undertriage. In addition, by avoiding excessive vital sign recording, the time spent on triage may have been influenced.

The hypothesis regarding the timing on electronic registration was also confirmed, showing a statistically significant difference between the two types of MTS application.

As for timing, there are few publications that quantify the triage timing using the MTS^(^ 41 ^)^. Some studies have found an average time of 1.45 and 4 minutes; however, the use of electronic or manual registration to perform the triage is not specified^(^ 22 ^,^ 42 ^)^.

However, a Portuguese study that analyzed data regarding the initial care of patients with chest pain from the electronic record obtained an average of 2 minutes of time spent with the triage^(^ 43 ^)^.

In the present study, the average time spent with triage on manual recording was slightly above the 3-minute time interval recommended by the GBCR^(^ 44 ^)^.

It can be assumed that the differences found in the time spent with the classification are due to the need for the individual to check guides or manuals during triage in phase 1 and be dependent on their memory^(^ 45 ^-^ 46 ^)^.

Electronic registration have the same information as the MTS book; however, users can access them quickly and directly with just a few clicks. In addition, the large number of errors related to vital sign recording, as discussed, may have made the manual process take longer.

A study describing the DSS shows that these systems are faster compared to the activities performed on paper^(^ 29 ^)^. And the shorter time spent on triage in the electronic registration can bring important advantages to the process.

Patients with a higher level of urgency will be evaluated in a shorter time and, consequently, referred sooner to medical care; those arriving at the emergency service will wait a shorter time in the waiting room for triage; the safety time for the classification (3 minutes) is respected.

Computerization is a reality in emergency services, for example, through systems for requesting and checking laboratory tests, online access to medical literature. Thus, DSS can improve the quality of care for emergency patients^(^ 45 ^)^.

The DSS make it possible to contribute to the work process of nurses, assisting in decision making, time optimization, accessibility and integration of information, as well as in the creation of indicators^(^ 29 ^,^ 47 ^-^ 48 ^)^.

The computerization of emergency care data and the construction of databases allow the analysis and comparison of care in different emergency services. Electronic registration makes it possible to measure the triage timing automatically; calculate the total number of patients treated in a given time frame, with real time updates; as well as identify the profile of the patients treated, both by their personal characteristics (gender, age) and those regarding the triage (flowchart, discriminator, priority). These data can help in health management, aiming at the quality, safety and humanization of emergency services.

Consequently, manual registrations do not allow updates, their content is available to only one person at a time, there are no backups and misinterpretations may occur due to the registration of unreadable letters^(^ 29 ^)^. Still, in manual records, often the absence of important data, such as date, time and identification of the professional, as well as errors and erasures, make it difficult to read and understand the records^(^ 5 ^)^.

This way, there are advantages with the use of electronic records for the application of MTS, assisting nurses in the decision-making process, minimizing the failures resulting from the absence or excess of records and providing less time spent in triage.

However, it is important to highlight the need for continuous improvement of health informatics professionals and the technological updating of electronic records, through the development of intelligent systems with patient complaint description algorithms, in order to support the decision-making process of nurses and contribute to the efficiency and effectiveness of the triage.

Some limitations of this study may be pointed out as the use of simulated cases, which do not consider possible interferences in the triage process in real situations, such as interruptions by patients or other employees, the nonverbal assessment of the patient, which could alter the triage results. The research took place in a single study center, which makes it difficult to compare with other units and to generalize the results obtained. And yet, the electronic record has alert signals that act as barriers to error, despite the prevailing clinical reasoning of professionals.

## Conclusion

The hypothesis was confirmed for reliability in choosing flowcharts and discriminators; for accuracy in determining flowcharts, discriminators and priority and for the timing to perform the triage.

The reliability regarding the priority variable was higher with the use of manual registration, however the values ​​obtained in both registrations reached at least moderate levels of agreement.

The electronic record showed higher reliability and accuracy for the vital signs variable and triage timing was significantly shorter.

Although it is possible to use both manual and electronic registration for triage, the results show greater advantages with the use of technologies in the management and care work process in the various health services.

It is also highlighted the importance of adopting content on triage and informatics in undergraduate nursing as a way to minimize the errors resulting from this process and to instruct professionals to use technologies. In addition, this knowledge will assist nurses in the interpretation of triage data, allowing its use as a collaborative tool for management, teaching and research.

Considering the scarcity of studies on the subject and its importance for the care in emergency services, further studies are suggested.

Despite the limitations mentioned, it was sought to use cases that are used by GBCR and are very close to the profile of patients treated at HU-USP.
